# Supplementary material for: Causal interplay between lactose intolerance and gut microbiota: a combined bidirectional Mendelian randomization and in vivo validation study
Source: Front Nutr. 2026 Jun 1;13:1803337. doi: 10.3389/fnut.2026.1803337 (PMC13265576; doi:10.3389/fnut.2026.1803337)
Supplement: Supplementary file 1 [file Data_Sheet_1.zip › supplementary materials/Reverse/Batch analysis results 20250616221646/leave one out cross validation/finngen_R12_E4_LACTONAS.ebi-a-GCST90027585.pdf]

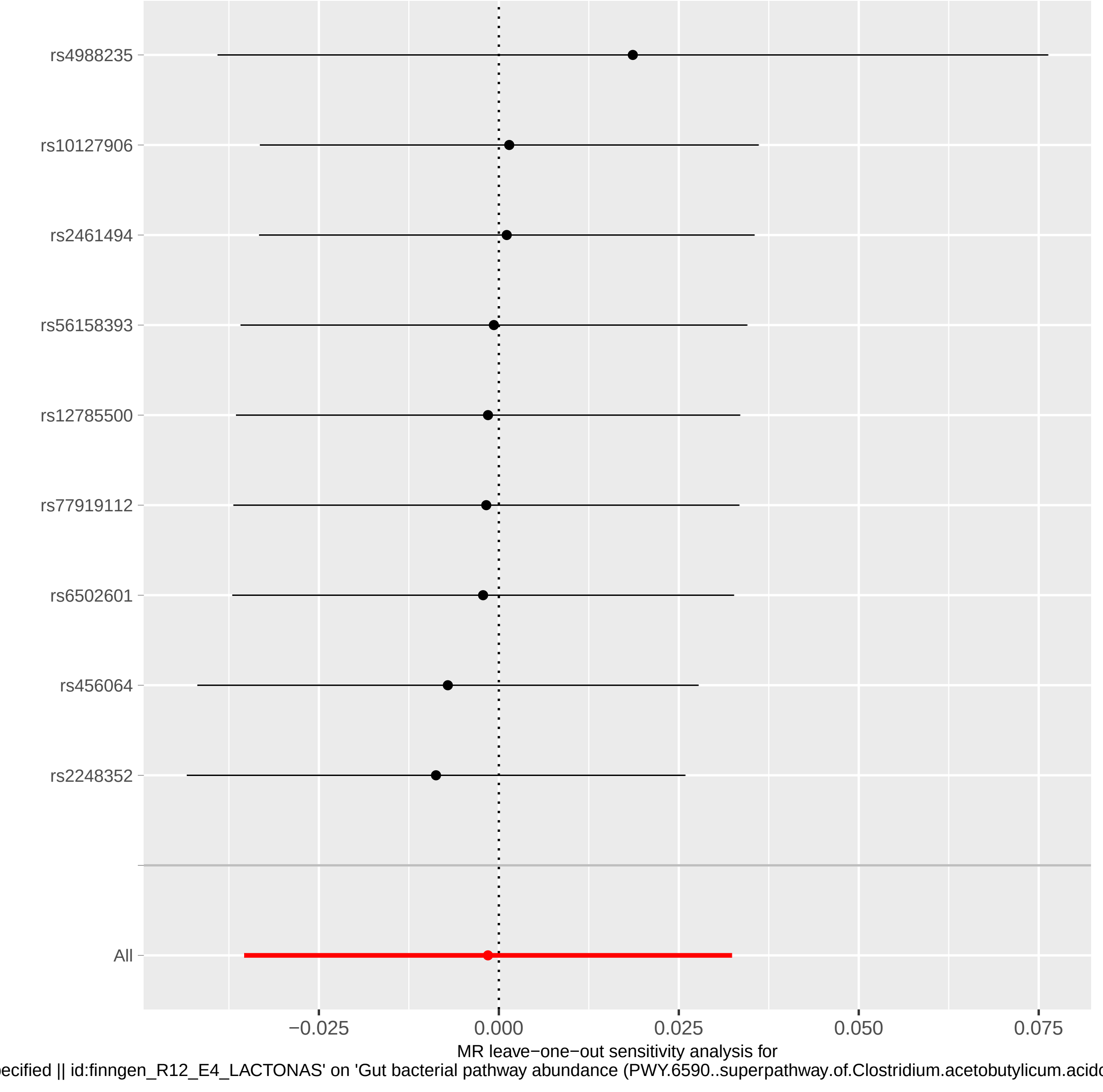
rs4988235
rs10127906
rs2461494
rs56158393
rs12785500
rs77919112
rs6502601
rs456064
rs2248352
All
−0.025
0.000
0.025
0.050
0.075
MR leave−one−out sensitivity analysis for
ecified || id:finngen_R12_E4_LACTONAS' on 'Gut bacterial pathway abundance (PWY.6590..superpathway.of.Clostridium.acetobutylicum.acido
